# Calcium Overload and in vitro Apoptosis of the C6 Glioma Cells Mediated by Sonodynamic Therapy (Hematoporphyrin monomethyl ether and ultrasound)

**DOI:** 10.1007/s12013-014-0081-7

**Published:** 2014-08-27

**Authors:** Dongning Hao, Yanbin Song, Zhen Che, Qi Liu

**Affiliations:** Neurosurgery Department, The First Hospital of Yulin, No. 59 of Suide Culture Road, Yulin city, 718000 Shanxi province China

**Keywords:** C6 Glioma cells, Sonodynamic therapy, Apoptosis, Calcium overload, ROS

## Abstract

The objective of this study was to investigate the role of intracellular calcium overload in the in vitro apoptosis of C6 glioma cells mediated by low level ultrasound and hematoporphyrin monomethyl ether (HMME) therapy. The frequency of ultrasound was optimized by the cell viability assay using 3-(4,5-dimethythiazol-2-yl)-2,5-diphenyltetrazolium bromide (MTT). The apoptotic rate, reactive oxygen species (ROS) and decreased mitochondrial membrane potential (MMP) were determined by flow cytometry. Morphological changes were observed by the transmission electron microscope. Concentrations of intracellular Ca2+, [Ca2+]i were detected by a confocal microscopic laser scanning, and the release of cytochrome-c (cyt-c) was measured by western blotting. Results: The SDT-mediated apoptotic effect involved an overload of [Ca2+]i derived from the intra- and extracellular sources during the early progression of apoptotosis. The process was associated with an increased ROS production, a decreased MMP, and a release of cyt-c. In conclusion,the combined use of low level ultrasound and HMME improved the apoptotic rate of C6 glioma cells mediated by ultrasound alone. The [Ca2+]i overload involving activation of mitochondrial signaling played a pivotal role in the SDT-induced apoptosis.

## Introduction

Because of an incomplete surgical resection and infiltration of the glioma cells, surgical treatment of this tumor results in a high rate of recurrence and metastasis. Various auxiliary therapies, including radiation, chemo, and photodynamic are used for the treatment of glioma ; however, the median survival rate remains far below the desired outcome [1]. The ultrasound has a unique ability to reach the internal targets by penetrating the intervening tissues and focus energy into small volumes. The porphyrins activated by ultrasound have been shown to significantly inhibit murine tumor growth. Hence, the use of photosensitive porphyrins for sensitizing the tumors to ultrasound treatment has been proposed and named as sonodynamic therapy (SDT) [1]. Previous studies have indicated that use of ultrasound with porphyrins induced apoptosis in glioma cells, however, with a relatively low rate [2–6]. Additional data have suggested that use of ultrasound of lower frequency and power improved the cavitation and concomitant biological effect [7–13]. In this study, we attempted to enhance the apoptosis effect in treating C6 glioma cells in vitro by using 10 ug/ml of hematoporphyrin monomethyl ether (HMME) and ultrasound with low frequency (0.5 MHz), and minimal intensity (1.0 W/cm^2^) for a duration of 60 s.

Apoptosis is controlled by many factors, such as reactive oxygen species (ROS), calcium overload, and mitochondrial damage [10–12]. Calcium ion has been suggested to play an important role in the regulation of apoptosis. An abrupt increase of intracellular Ca2+, [Ca2+]i is known to occur in various conditions including ischemia–reperfusion injury, receptor over-stimulation, oxidative stress,and so on [13–16]. Calcium overload leading to mitochondrial damage causes release of apoptotic promoters and activation of the caspase cascade [14–16]. Honda et al. [17] found that a treatment of human myelomonocytic lymphoma U937 cells with ultrasound alone caused a transient increase in [Ca2+]i. Li et al. [18] showed that C6 glioma cells treated with 1 MHz ultrasound and HMME led to an increased [Ca2+]i released from internal stores. However, the mechanism of increased [Ca2+]i caused by the combined use of low level ultrasound and HMME and its effect on apoptosis has not been completely elucidated.

Accumulating evidence has demonstrated that the SDT-induced apoptosis of C6 glioma cells is mediated through an excessive production of ROS incurred by the interaction of the ultrasonic cavitation and the sensitizers [7, 13]. The oxidizing damage affects mitochondria and leads to the activation of a cascade of apoptosis promoting signaling pathway [13–16]. In addition, in the absence of an extracellular source, ROS has been found to increase the cytosolic calcium, leading cells into apoptotic state [17]. Cavitations, including inertial and stable, affect many biological processes, such as production of the free radicals, change of membrane permeability, and sonoluminescence [14–17]. The mechanism of ROS production has not been clearly understood. Nonetheless, the cavitation effect involved in the SDT-mediated apoptosis may be assumed to cause mitochondrial damage through the overloaded Ca2+ and thereby lead to apoptosis.

Accordingly, we hypothesized that treatment of the C6 glioma cells with low level ultrasound in combination with HMME might promote apoptosis by increasing the concentration of [Ca2+]i. It is also possible that increased levels of [Ca2+]i may trigger the production of ROS, decrease mitochondrial membrane potential (MMP), and release of cytochrome c (cyt-c). We determined the apoptotic rate and the concentration of [Ca2+]i in SDT- treated glioma cells. The L-type Ca2+ channel antagonist nimodipine was used to find out as to whether the source of [Ca2+]i overload was extracellular.

## Methods

### Regents

The C6 glioma cell line was purchased from the Beijing Institute of Biology, Chinese Academy of Science (Beijing, China); 2,7-dichlorodihydrofluorescein diacetate (DCFH-DA), Rhodamine 123 and fluo-3/acetoxymethylester were purchased from Sigma (St Louis, MO). FITC-Annexin-V/PI and HBSS were purchased from Beyotime (Jiangsu, China). Nimodipine was purchased from Bayer (Germany). HMME was purchased from Kangmei Chemical Co. Ltd. (Shenzhen,China). Polyclonal antibodies against cyt-c were from purchased from Santa Cruz (San Diego, CA).

### Cell Culture

The C6 glioma cells were cultured in RPMI 1640 containing 10 % fetal bovine serum (FBS). The cells were maintained at 37 °C in a humidified atmosphere containing 5 % CO2. Once grown to 70–80 % confluence, the cells were trypsinized, counted, and seeded in 6-well plates at a density of 1 × 106/ml cells per well for the subsequent experiments.

### Ultrasound Frequency Optimization

To optimize the ultrasound frequency, the cell viability assay using 3-(4,5-dimethythiazol-2-yl)-2,5-diphenyltetrazolium bromide (MTT, Sigma) was carried out as described previously [16]. Cells were cultured at 37 °C in 6-well plates at a density of 1 × 106/ml cells per well. The ultrasound treatment was carried out at room temperature in a sponge water bath (depth of 10 mm) using a multi-function ultrasound device (Selfridge, Beijing; ultrasound transducer diameter 20 mm, depth of 50 mm penetration). The sponge was placed under the wells, and the probe was under the sponge. The sponge water bath helped to minimize the acoustic reflections and subsequent standing wave formations. The pulsed-wave ultrasound with a low intensity (1 W/cm^2^) was applied for a duration of 60 s. The ultrasound frequencies varied from 0 to 1.0 MHz. After this treatment, the cells were trypsinized and transferred to 96-well plates and incubated with 0.5 mg/ml of MTT (final concentration) at 37 °C for 4 h. Then the medium was removed and 200 μl of dimethylsulfoxide (DMSO, Sigma) were added. The absorbance was read at 490 nm with a universal microplate spectrophotometer (BIO-RAD Model 550). The cell viability at 0 MHz was considered as control, and the others were expressed as percentage of control. Cell viability was statistically analyzed to select the optimal frequency for subsequent ultrasound treatments.

### Cell Treatment

The ultrasound and SDT treatment of the C6 glioma cells were conducted as previously described [6]. Briefly, cells cultured at 37 °C in six-well plates were randomly divided into control (untreated), HMME (HMME alone), ultrasound (ultrasound alone) and SDT (ultrasound plus HMME) groups. Each group consisted of six wells. Cells in these four groups were pretreated with Ca2+-free PBS or HBSS (containing 1.3 mM Ca2+, pH 7.4). Nimodipine was used at a final concentration of 10 μM for 2 h before insonation. Then the cells incubated in different reagents were treated with an instant insonation of the same strength and duration, however, various frequencies. After treatment, the cells were maintained at 37 °C in a dark, humidified atmosphere containing 5 % CO2 for the subsequent experiments.

### Measurement of Intracellular Ca2+

The concentration of intracellular calcium was measured by confocal laser scanning microscopy as described previously [11]. The C6 glioma cells cultured in 96-well plates were incubated with 10 μM fluo-3/acetoxymethylester at 37 °C for 30 min. Cells were then rinsed three times with PBS to remove the extracellular fluo-3/acetoxymethylester and then SDT was applied. For measurement of the [Ca2+]i, fluorescence imaging was carried out at the excitation and emission wavelengths of 488 nm and 530 nm ,respectively, using a confocal laser scanning microscope for 1800 s (Leica, Germany).

### Analysis of Apoptosis

Cell apoptosis was measured by flow cytometry after double staining of the cells with FITC-Annexin-V and PI as previously described [11]. After 24 h, cells receiving different treatments were harvested, washed twice with phosphate buffered solution (PBS) and re-suspended in 0.5 ml PBS at a cell density of 1 × 106/ml. Annexin-V (5 μl) and 10 μl PI were added to the wells in the dark. After 30 min of incubation, the apoptotic rate was determined by flow cytometry (Becton–Dickinson, USA).

### Transmission Electron Microscopy

After 24 h of the treatments, the cells were harvested and fixed with 3.0 % glutaraldehyde and 1.5 % paraldehyde, washed in PBS, and fixed in osmium tetroxide. Then, they were dehydrated with a series of increasing concentrations of ethanol, embedded in epoxy resin, and examined under the transmission electron microscope (JEM-1220EX).

### Production of Intracellular ROS

The production of intracellular ROS was assayed by flow cytometry using DCFH-DA as previously described [6]. After 2 h of treatments, the cells were harvested, washed, and re-suspended in 500 μL PBS containing DCFH-DA (final concentration 50 μM) and incubated at 37 °C in the dark for 30 min. The flow cytometry was then performed.

### MMP Detection

The loss of MMP was detected using Rhodamine 123 and flow cytometry as previously described [6]. After 2 h of various treatments, Rhodamine 123 was added to the cells with a final concentration of 10 μg/mL in the dark and incubated for 30 min. The loss of MMP was calculated using CELLQuest software.

### Detection of Cytochrome-c Release From Mitochondria

To quantify cyt-c release, western blot analysis of the cytosolic fraction was performed as previously described [19]. Briefly, cells after treatments were harvested, washed twice with ice-cold PBS, and incubated in an ice-cold Tris-sucrose buffer (0.35 M sucrose, 10 mM Tris–HCl, pH 7.5, 1 mM EDTA, 0.5 mM dithiothreitol, 0.1 mM phenylmethylsulfonyl fluoride). After a 40 min incubation, cells were centrifuged at 1,000×*g* for 5 min at 4 °C, and the supernatant was further centrifuged at 40,000×*g* for 30 min at 4 °C. The supernatant was retained as the cytosolic fraction and analyzed by Western blotting using a primary anti- cyt-c monoclonal antibody and then a secondary antibody (Santa Cruz). Actin expression was used as the control.

### Statistics

Data were expressed as mean ± SEM. Comparisons in different groups were performed with factorial design analysis of variance (ANOVA) by SPSS software 11.0. *P* < 0.05 was considered statistically significant.

## Results

### Optimal Ultrasound Parameters

**Fig. 1:**
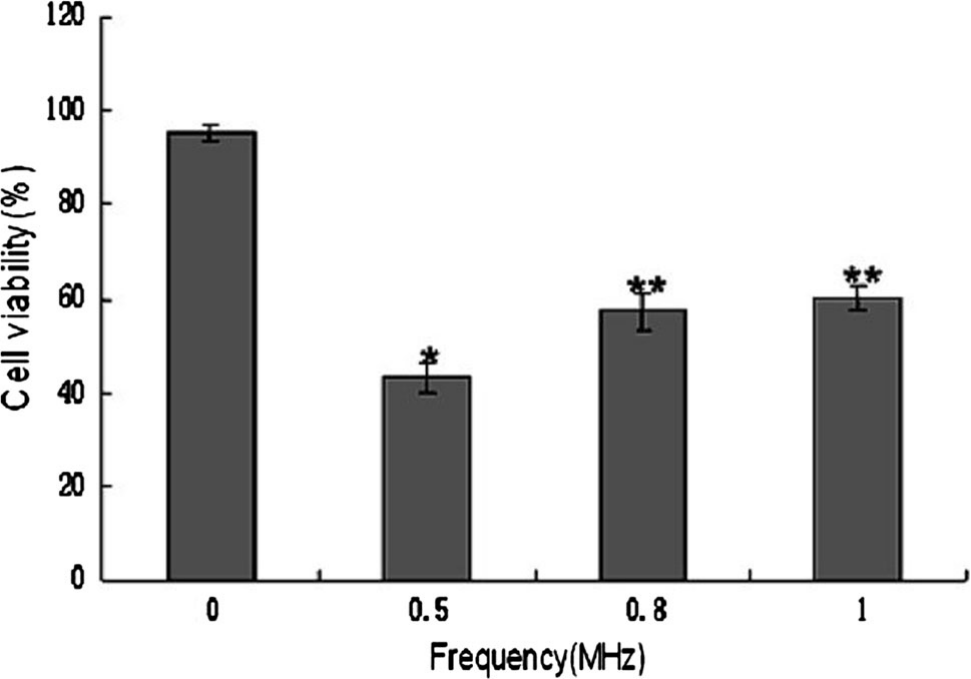
The viability of C6 glioma cells after treatment with ultrasound of under 1.0 W/cm^2^ for 60 s at frequencies varying from 0 to 1.0 MHz. Compared to the controls, the significant decrease (*P* < 0.05) in viability caused by 0.05 MHz and 0.8 MHz or 1.0 MHz are represented by * and ** respectively. Data are expressed as mean ± SEM from six independent experiments

Cells were exposed to pulsed-wave ultrasound with an average power of 1.0 W/cm^2^ applied for 60 s. The ultrasound frequency was optimized by the cell viability assay using MTT. Figure 1 shows that survival rates were 95.4 ± 1.8, 43.2 ± 3.2, 57.1 ± 3.7 and 60.2 ± 2.6 % after insonation with 0, 0.5, 0.8 and 1 MHz frequencies , respectively. Since the survival was decreased with 0.5 MHz insonation, this frequency was used in the subsequent treatment experiments to improve the apoptotic rate of C6 glioma cells.

### Measurement of [Ca2+]i

**Fig. 2:**
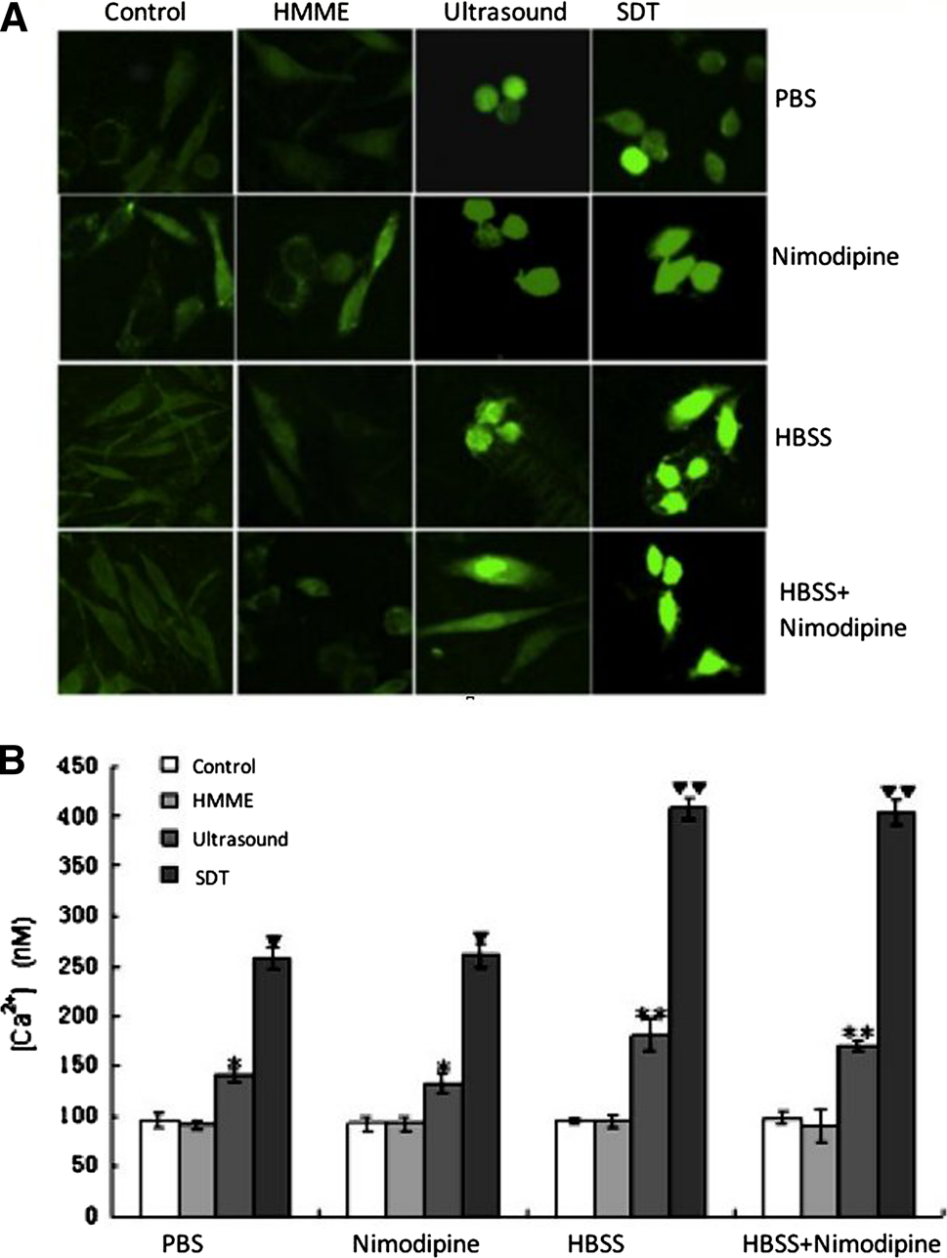
Effect of nimodipine on the change in intracellular free calcium induced by various treatments. **a** Fluorescent images of C6 glioma cells, **b** Quantification of [Ca2+]i in different treatment groups. The cells incubated in PBS or HBSS with and without nimodipine were treated with HMME, ultrasound or SDT and imaged by confocal laser scanning microscopy in 1800 s. P < 0.05 was considered as significant. In PBS group:Increase was significant comapared to the controls*, compared to the response of ultrasound alone▼, HBSS group: ** significant compared to the HBSS control, ▼▼significant compared to the ultrasound treatment response

The concentration of intracellular free calcium was measured in 1800 s by confocal laser scanning microscopy in a single cell using fluo-3/acetoxymethylester, the fluorescent probe sensitive for Ca2+. The concentration of [Ca2+]i was significantly increased in the cells treated with ultrasound in PBS or HBSS, with or without nimodipine. In the cells treated with SDT, the concentration of [Ca2+]i was further enhanced and it was statistically significant (*P* < 0.05) compared to the controls (Fig. 2a, b). These observations suggest that the [Ca2+]i overload was mediated by SDTtreatment of the cells. The SDT treatment of the cells caused an elevation of [Ca2+]i concentration in 1800 s (258 ± 11.8 nM) and compared to the controls in calcium-free PBS, it was significant (*P* < 0.05). The level of [Ca2+]i was observed to be further elevated with SDT treatment of the cells in HBSS containing calcium (408 ± 11.6 nM). Compared to the controls, it was significant (*P* < 0.05). Also, addition of nimodipine to HBSS was not found to augment the SDT-mediated concentration of [Ca2+]i.

### Overload of [Ca2+]i and Apoptotic Effect

**Fig. 3:**
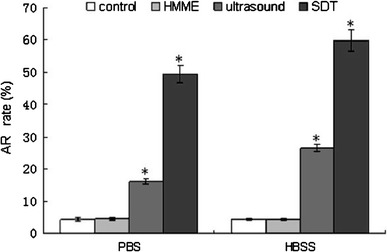
Apoptotic rate (AR) of the C6 glioma cells mediated by HMME, ultrasound and SDT, and ultrasound treatment in calcium-free PBS or HBSS measured by FCM. Compared to control, the increase was considered significant when *P < 0.05

To demonstrate the relation between apoptosis and overloaded [Ca2+]i, apoptotic rates were determined by flow cytometry in the cells that exhibited overloaded [Ca2+]i. Cells were untreated (controls) or treated with HMME, ultrasound, and SDT in calcium-free PBS or in HBSS containing calcium. The SDT and ultrasound treatments in HBSS showed a significant elevation (*P* < 0.05) in apoptotic rate compared to that in PBS (Fig. 3). The ultrasound-mediated apoptosis was 26.5 ± 1.1 and 16.0 ± 0.83 % in HBSS and PBS ,respectively. The SDT treatment in HBSS or calcium-supplemented medium showed further and significant increase in apoptosis (49.4 ± 2.6 %).

### Morphological Changes

**Fig. 4:**
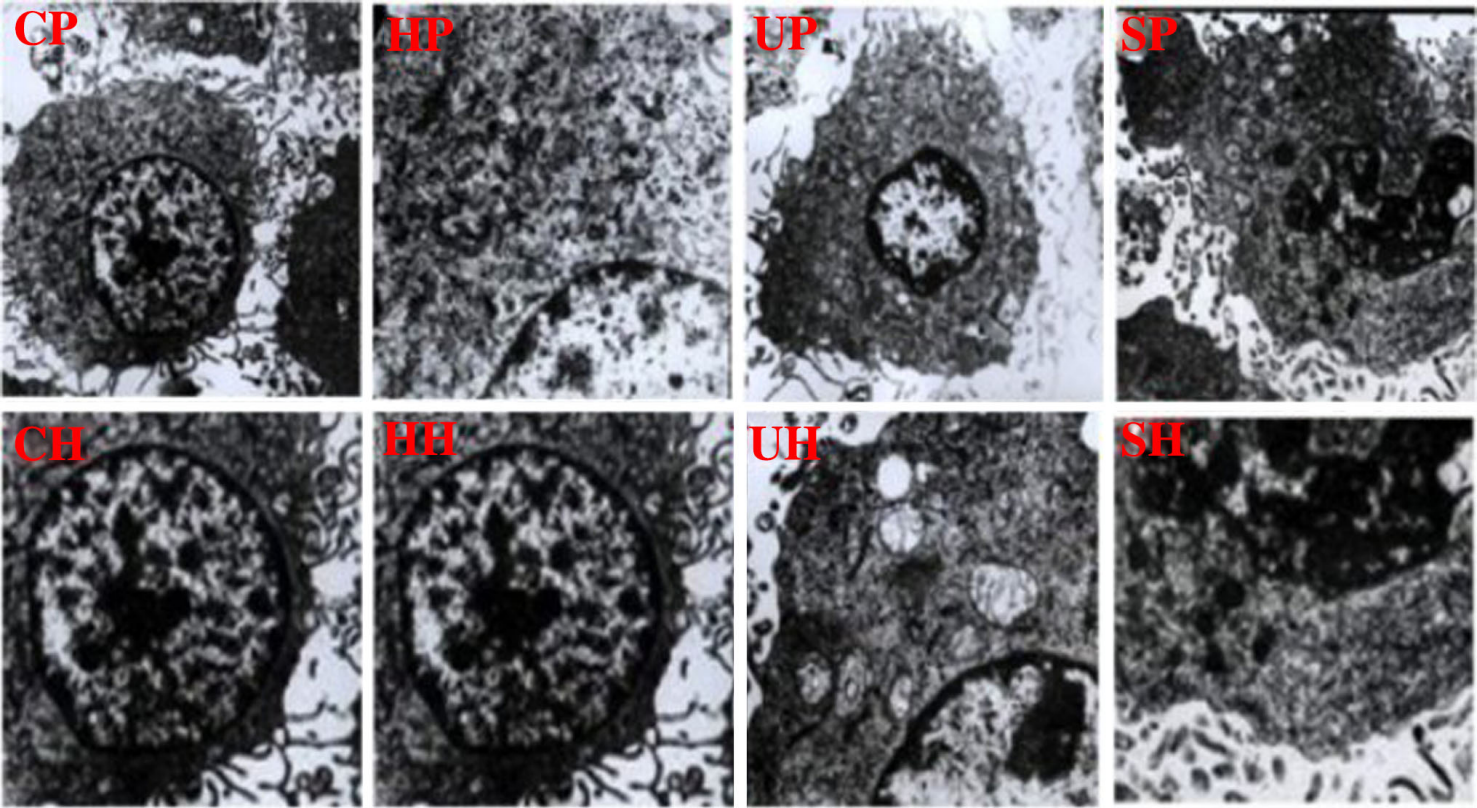
TEM images (magnification × 10,000) of ultrastructural changes in C6 glioma cells treated with PBS (control) (CP), HMME-PBS (HP), ultrasound-PBS (UP), SDT-PBS (SP), HBSS (control) (CH), HMME-HBSS (HH), ultrasound-HBSS (UH) and SDT-HBSS (SH)

The apoptotic effect of SDT treatment was also determined by the morphological changes in C6 glioma cells observed by transmission electron microscopy. Cells treated with ultrasound and SDT in either PBS or HBSS showed margination of nuclear chromatin, aggregation, condensation, mitochondria swelling, and vacuolization. Whereas the cells treated with HMME in either PBS or HBSS exhibited intact cell membrane and nuclear envelope, rich cytoplasm, and integrated mitochondria (Fig. 4).

### Intracellular ROS

**Fig. 5:**
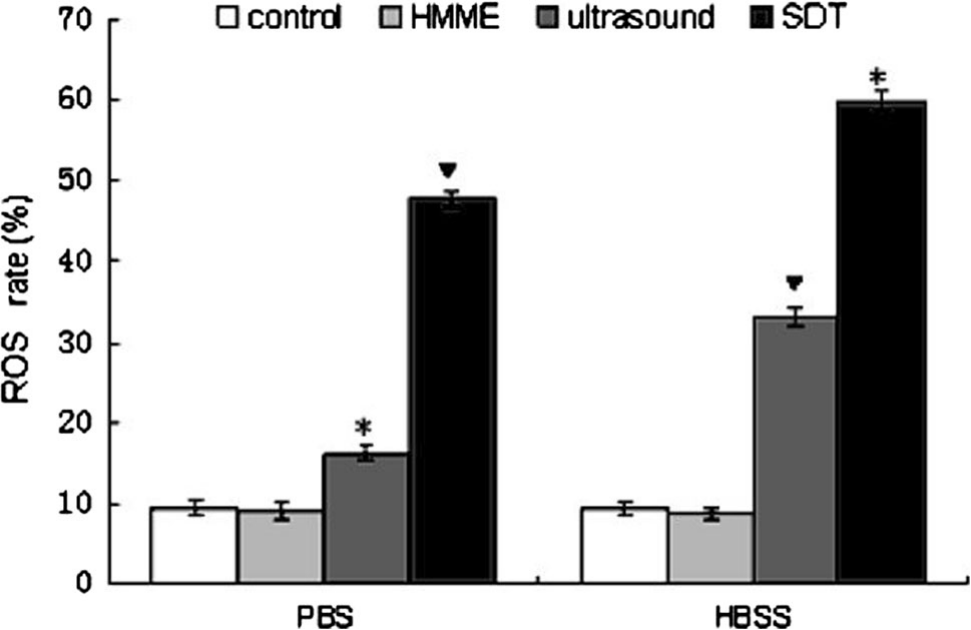
ROS production by the C6 glioma cells treated with HMME, ultrasound, and SDT in calcium-free PBS or HBSS, measured by FCM. Compared to the controls, the SDT-treated cells showed significantly increased (*P* < 0.05) production of ROS group. *Asterisk* represents increase compared with the control groups.▼represents increase compared to ultrasound treated group

The production of ROS determined by flow cytometry following incubating the cells with DCFH-DA was found to be significantly increased (*P* < 0.05) by ultrasound and SDT treatment (Fig. 5). The ultrasound and SDT-treated cells in PBS produced 16.1 ± 1.0 and 47.7 ± 1.2 % ROS ,respectively. Whereas these treatments in HBSS generated 35.5 ± 1.0 and 60 ± 1.2 % ROS ,respectively. The trend of SDT-induced augmentation of the ROS generation in calcium-supplemented medium coincided with the increase of apoptotic rate and [Ca2+]i overload.

### Loss of MMP

**Fig. 6:**
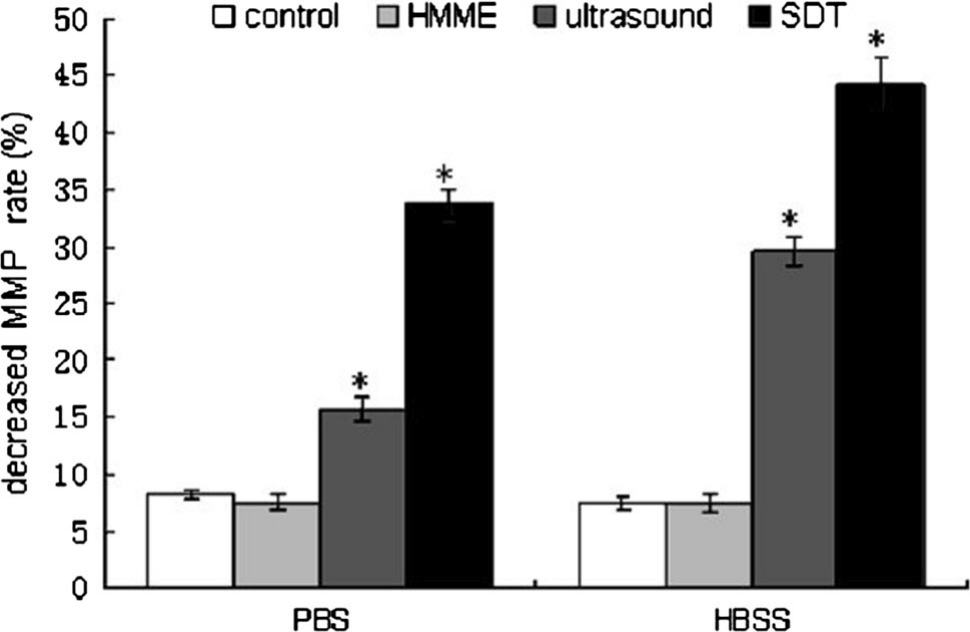
Effect of calcium in the extracellular medium on MMP reduction in the C6 glioma cells treated with HMME, ultrasound and SDT. The MMP was measured by FCM. The effect was considered significant when *P < 0.05

Mitochondria of the cells undergoing apoptosis normally lose their membrane potential that appears as a reduced fluorescence produced by rhodamine123. Thus, MMP determined by flow cytometry after staining the cells with rhodamine123. Compared to the control cells, the ultrasound-treated cells exhibited a significant reduction (*P* < 0.05) in MMP (Fig. 6). The SDT treatment of the cells further decreased the MMP, particularly in HBSS, the calcium-supplemented medium. The trend of SDT-induced MMP loss was found to be parallel to the increased ROS and [Ca2+]i overload in the cells undergoing apoptosis by this treatment.

### Release of cyt-c

**Fig. 7:**
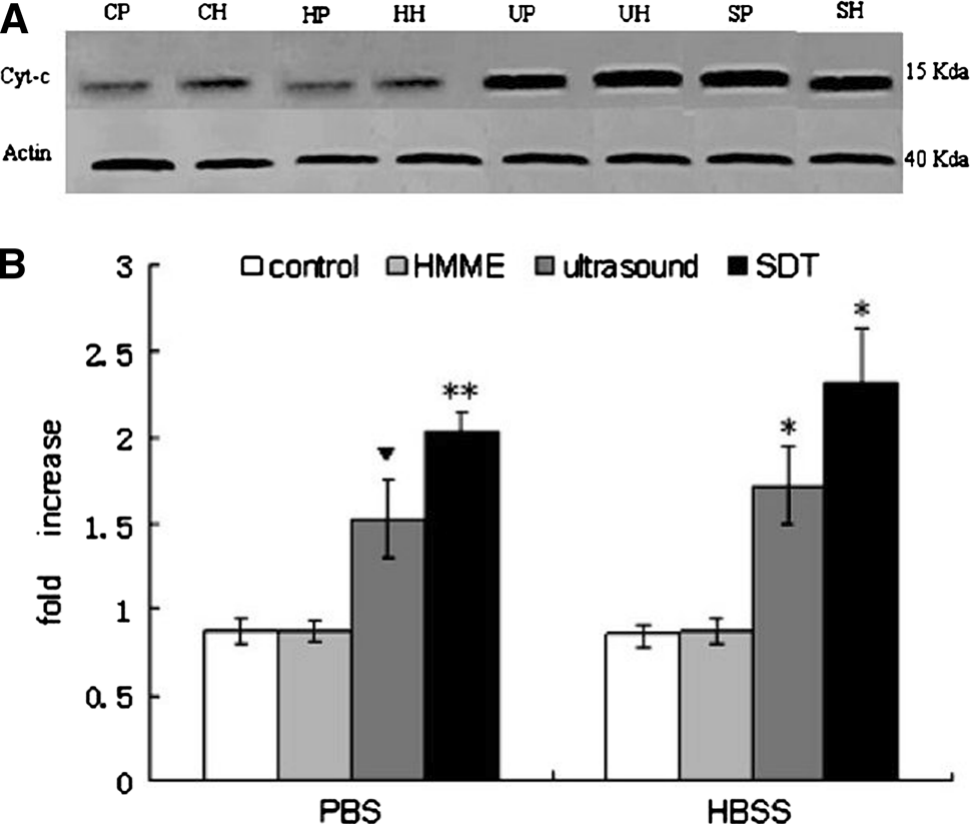
Western blot analysis showing the release of cyt-c from HMME, ultrasound and SDT treated C6 glioma cells incubated either in calcium-free PBS or HBSS. A. Western blot, B. Quantification of the released cyt-c showing fold increase by various treatments. Compared to the control, the effect was significan when *P < 0.05

Since MMP reduction in apoptotic cells leads to the release of cyt-c from the mitochondria, we measured the levels of this protein by Western blotting in the SDT-treated cells. The release of cyt-c was found to be significantly (*P* < 0.05) up-regulated in the cells treated with ultrasound or SDT in both PBS and HBSS (Fig. 7). The release of cyt-c from the cells treated with HMME alone either in PBS or HBSS was not significantly increased (*P* > 0.05).

## Discussion

The apoptotic effect of SDT has been reported to depend on ultrasound intensity, frequency, duration, sonosensitizers, etc. [20]. Usually, powers below 3 W/cm^2^ are considered as low [21, 22], and the frequencies under 1 MHz are commonly used for drug delivery, opening of the blood–tumor barrier (BTB) and ultrasonic therapy [23]. Buldakov et al. [23] observed apoptosis in U937 cells treated with ultrasound power of 0.3 W/cm^2^, and frequency of 1 MHz. They suggested that lower the frequency and intensity of ultrasound is, more the cavitation and consequent biological effect would be [7, 23]. In the present study, we treated the C6 glioma cells with HMME and the optimized ultrasound intensity and frequency of 1.0 W/cm^2^, and 0.5 MHz, respectively, for a duration of 60 s. The treatment showed occurrence of apoptosis detected by flow cytometry and transmission electron microscopy. The apoptosis rate was found to be significantly increased to 49.4 ± 2.6 and 59.9 ± 3.2 % in the SDT-treated cells in PBS or HBSS ,respectively. Clearly, an improvement over the previous studies showing less than 40 % apoptosis in C6 glioma cells [3, 6, 18, 20]. These results suggest that low level ultrasound combined with HMME may improve the C6 glioma cells apoptosis.

Calcium ions play a pivotal role in the regulation of cell proliferation and death [15]. Thus, maintenance of intracellular Ca2+ homeostasis is crucial for the normal cellular functioning [16]. Within the cell, Ca2+ gradient is also established between the cytoplasm and the cell organelles, such as the endoplasmic reticulum (ER) and mitochondria [17]. A change in Ca2+ homeostasis could influence the ultimate fate of the cells. An overload of [Ca2+]i is known to alter the mitochondrial membrane permeability facilitating release of cyt-c and other apoptotic factors, and thereby promotes apoptosis [10–12]. Our results showed that the concentration of [Ca2+]i was significantly elevated in the SDT-treated cells pre-incubated in Ca2+-free PBS, indicating the role of [Ca2+]i overload in this treatment. In order to find out if the source of [Ca2+]i overload, was intra or extracellular, prior to the SDT treatment, the cells were incubated in either Ca2++-free PBS or HBSS buffer that contained 1.3 mM Ca2+. The results showed that SDT treatment caused more increase in the [Ca2+]i concentration in HBSS pretreated-cells, suggesting the contribution of Ca2+ influx from the extracellular source. An increased [Ca2+]i derived from both intra and extracellular sources, in the early phase of apoptosis has been suggested by other studies [12, 19, 22]. However, increased [Ca2+]i either from intra or extracellular source has also been reported previously [17, 18, 24, 25]. The discrepancy may be attributed to the difference in ultrasound parameters and the types of cells used. As proposed by Liu et al. [4, 26, 27], the mechanisms of SDT treatment are influenced by multiple factors, such as, nature of the biological model, the sonosensitizer, and the ultrasound parameters, etc. The lack of nimodipine effect on [Ca2+]i concentration in the cells incubated in HBSS prior to SDT treatment suggests that L-type voltage-dependent calcium channels were not involved in the Ca2+ influx. Therefore, it is proposed that the Ca2+ influx may be mediated through ways other than voltage-dependent Ca2+ channels, such as promotion of membrane permeability, and perforation [21]; however, that remains to be confirmed. Furthermore, our results revealed that the maximal [Ca2+]i in the SDT-treated cells corresponded to the highest rate of apoptosis. Clearly suggesting that level of [Ca2+]i overload is directly related to the apoptotic rate induced by SDT treatment. Also, the increase in [Ca2+]i contributing to SDT-mediated apoptosis was acquired from both intra and extracellular sources.

Whereas at lower concentrations, ROS functions as a mediator of intracellular signaling, at the higher levels, it could cause toxic effects, such as lipid peroxidation and induced cell death without an involvement of the caspase cascade [11]. Generation of ROS in situ can promote the permeability of mitochondrial membrane with subsequent decrease in MMP. As a result of decreased MMP in the early stage of apoptosis, cyt-c, AIF, and Smac/Diabio are released from mitochondria into the cytosol. Then Caspase-9 and-3 are activated to carry out the irreversible process of apoptosis [12, 19, 22]. Although mitochondria are known to mediate Ca2+ buffering by sequestering the excessive calcium from cytosole, an excessive calcium load may impair the permeability of their membrane and thereby stimulate the release of factors that promote apoptosis. Previous studies have shown that SDT-induced apotosis was correlated with the ROS production and increased permeability of mitochondrial membrane allowing release of apoptotic molecules into the cytosol [3, 16]. The results obtained in the present study showed an enormous increase in ROS production, a decreased MMP, and release of cyt-c from the cells treated with ultrasound and SDT in the media supplemented with or free of Ca2+. Clearly, these results suggest the involvement of mitochondrial signaling in the apoptotic process. In addition, we observed that ROS production was directly correlated to the increasing [Ca2+]i concentration. In conclusion, the SDT (low strength ultrasound combined with HMME) treatment of the cells increased [Ca2+]i, that caused enhanced ROS production which in turn decreased the MMP and that led to the release of cyt-c to promote apoptosis. The treatment of the C6 glioma cells with the current dosage of SDT showed an improved outcome compared to that reported by the previous studies.

In conclusion, the use of low strength ultrasound along with HMME led to an augmentation of the C6 glioma cells apoptosis. Compared to the previous studies, the effect was improved and it was mediated through enhancing the ROS production by promoting [Ca2+]i flux from both extra and intracellular sources. Furthermore, an impairment of MMP accompanied with increased cyt-c release suggests the contribution of mitochondrial signaling in the apoptotic effect of SDT.
